# Ligase IV inhibitor SCR7 enhances gene editing directed by CRISPR–Cas9 and ssODN in human cancer cells

**DOI:** 10.1186/s13578-018-0200-z

**Published:** 2018-02-19

**Authors:** Zheng Hu, Zhaoying Shi, Xiaogang Guo, Baishan Jiang, Guo Wang, Dixian Luo, Yonglong Chen, Yuan-Shan Zhu

**Affiliations:** 1 Department of Clinical Pharmacology, Xiangya Hospital, Central South University, Changsha, 410078 Hunan China; 2 Translational Medicine Institute, National and Local Joint Engineering Laboratory for High-through Molecular Diagnosis Technology, The First People’s Hospital of Chenzhou, Chenzhou, 432000 Hunan China; 3 Department of Biology, Guangdong Provincial Key Laboratory of Cell Microenvironment and Disease Research, Shenzhen Key Laboratory of Cell Microenvironment, Southern University of Science and Technology, Shenzhen, 518055 Guangdong China; 4 Key Laboratory of Regenerative Biology, South China Institute for Stem Cell Biology and Regenerative Medicine, Guangzhou Institutes of Biomedicine and Health, Chinese Academy of Sciences, Guangzhou, 510530 Guangdong China; 5 Institute of Chemical Biology, Guangzhou Institutes of Biomedicine and Health, Chinese Academy of Sciences, Guangzhou, 510530 Guangdong China; 6 Department of Medicine, Weill Cornell Medical College, New York, NY 10065 USA

**Keywords:** CRISPR/Cas9, Homology-directed repair, DNA ligase IV inhibitor, Single-stranded oligodeoxynucleotides (ssODN), Non-homologous DNA end joining

## Abstract

**Background:**

Precise genome editing is essential for both basic and translational research. The recently developed CRISPR/Cas9 system can specifically cleave a designated site of target gene to create a DNA double-strand break, which triggers cellular DNA repair mechanism of either inaccurate non-homologous end joining, or site-specific homologous recombination. Unfortunately, homology-directed repair (HDR) is challenging due to its very low efficiency. Herein, we focused on improving the efficiency of HDR using a combination of CRISPR/Cas9, eGFP, DNA ligase IV inhibitor SCR7, and single-stranded oligodeoxynucleotides (ssODN) in human cancer cells.

**Results:**

When Cas9, gRNA and eGFP were assembled into a co-expression vector, the disruption rate more than doubled following GFP-positive cell sorting in transfected cells compared to those unsorted cells. Using ssODNs as templates, SCR7 treatment increased targeted insertion efficiency threefold in transfected cells compared to those without SCR7 treatment. Moreover, this combinatorial approach greatly improved the efficiency of HDR and targeted gene mutation correction at both the GFP-silent mutation and the β-catenin Ser45 deletion mutation cells.

**Conclusion:**

The data of this study suggests that a combination of co-expression vector, ssODN, and ligase IV inhibitor can markedly improve the CRISPR/Cas9-directed gene editing, which should have significant application in targeted gene editing and genetic disease therapy.

**Electronic supplementary material:**

The online version of this article (10.1186/s13578-018-0200-z) contains supplementary material, which is available to authorized users.

## Background

To generate sophisticated genetic modifications of endogenous genes is a valuable technology in the study of developmental biology, disease pathogenesis, and gene therapy of monogenic diseases. Traditional gene targeting, based on homologous recombination using the gene-targeting vectors with long homologous sequences, has revolutionized the field of mouse genetics and is suitable for generating sophisticated genetic modifications in endogenous genes [1]. However, this modification is laborious and time-consuming. DNA double-strand breaks (DSBs) induced by site-specific nucleases can stimulate homologous recombination in mammalian cells [2–4]. The engineered sites-specific nucleases, zinc-finger nuclease (ZFN) and transcription activator-like effector nuclease (TALEN) have been successfully applied in various animal models and mammalian cells for genome editing [5]. Recently, the CRISPR–Cas9 system, a new class of genome editing tool based on clustered regularly interspaced short palindromic repeats (CRISPR) and Cas9 protein, has been established, which, through an engineered single-guide RNA (sgRNA), directs Cas9 to the target sites by base-pair complementarity between the sgRNA and a target genomic DNA sequence for destruction targeted sequence [6, 7]. Due to easy handling, the CRISPR–Cas9 system has been applied immediately and widely to targeted genome modifications in vivo and in vitro [7–22].

Non-homologous end-joining (NHEJ) and homologous recombination are two major pathways for repairing DSBs. NHEJ is template-independent and directly joins the two DNA ends via DNA ligases, often leading to insertion/deletion (indel) mutations in double-strand break loci [23]. Homologous recombination is a DNA metabolic process found in all forms of life and provides a high-fidelity and template-dependent repair of DSBs [24]. Both DNA fragments or plasmids and single-stranded oligonucleotides (ssODN) with identical or nearly identical sequences can be used as templates to repair site-specific DSBs in mammalian cells [15, 25–27]. These two repair pathways appear to compete each other for DSBs [28], and a blockade of the NHEJ pathway facilitates homology-directed repair (HDR) in both in vivo [29–31] and in vitro systems [2, 32–36].

As the activity of DNA ligase IV is required for NHEJ pathway, increased HDR efficiency has been achieved in vivo and in vitro through using either a dominant-negative form of ligase IV or ligase IV-specific siRNAs to downregulate ligase IV activity [29, 30, 32]. In the current report, we have demonstrated an improvement of HDR and gene mutation correction efficiency using a combination of modified CRISPR/Cas9 co-expression vectors, ssODN as templates and ligase IV inhibitor SCR7 treatment in human cancer cells.

## Methods

### Oligonucleotides, primers and ligase IV inhibitor

All ssODNs used for transfection studies were manufactured by GenScript (Nanjing, China). Primers used for PCR and oligonucleotides used for annealing were synthesized by GIGA Biotechnology (Guangzhou, China). The ligase IV inhibitor, SCR7, was purchased from Xcess Biosciences Inc. (San Diego, CA) or synthesized as the procedure described by Srivastava et al. [37]. The endonucleases were obtained from New England Biolabs Inc. (Ipswich, MA, USA), and DNA purification kits were purchased from Tiangen Co. (Beijing, China).

### Construction of pCS2-Cas9-IRES-GFP-polyA-U6-sgRNA

The construction of the Cas9 expression vector was performed as we previously described [16]. Briefly, to create a sgRNA expression vector, we placed a U6 promoter followed by two BbsI sites upstream of the recently described sgRNA scaffold [7], which was synthesized by GenScript and cloned into the pUC57-Simple vector [16]. The sgRNAs were designed to target sequences in genes of interest with the form of 5′-G-(N)19-NGG-3′ [7]. The locus-specific 20-basepair protospacer containing the cloning cohesive sites was obtained by annealing two synthesized partially complementary oligonucleotides, and then cloned into the BbsI-digested gRNA expression vector. To construct a Cas9 and sgRNA co-expression vector, the IRES-eGFP sequence was inserted into the locus in front of PolyA of the pCS2-3 × FLAG-NLS-SpCas9-NLS-PolyA vector, and a pair of primers with the cloning cohesive sites *Xho*I and *Xba*I (Additional file 1: Table S1) was used to amplify the U6-sgRNA expression frame from pUC57-U6-sgRNA. The U6-sgRNA fragment was then cloned into pCS2-3 × FLAG-NLS-SpCas9-NLS-polyA-IRES-eGFP to generate the vector pCS2-3 × FLAG-NLS-SpCas9-NLS-IRES-eGFP-polyA-U6-sgRNA (pCS2-Cas9-IRES-GFP-polyA-U6-sgRNA) (Additional file 1: Figure S1).

### Lentivirus packaging and stable clone establishment

For evaluation of mutation correction efficiency in MCF-7 cells, a silent mutation was created in GFP ORF sequence (GFP-Mut) with a replacement of AC to GA at position 118 and 119 of GFP ORF (Additional file 1: Figure S3). The GFP-Mut or the GFP-Wild fragments were then cloned into lentivirus vector pSIN-EF1-IERS-Puromycin. Either the GFP-Mut lentivirus vector, pSIN-EF1-GFP-Mut-Puromycin, or GFP-Wild lentivirus vector was cotransfected with auxiliary pSPAX2 and pMD2.G plasmids to 293T cells to generate lentivirus. Following lentivirus infection, MCF-7/GFP-Mut cell clones were screened by puromycin and positive cell clones were used for the experiments.

### Cell culture, cell transfection, cell treatment and cell sorting

The human cell lines MCF-7, HCT-116, and K562 were obtained from American Type Culture Collection (ATCC, Manassas, VA, USA). HCT-116 cells were grown in McCoy’s 5A medium supplemented with 10% fetal bovine serum (FBS), 1.5 mM l-glutamine, 100 μg/ml streptomycin, and 100 U/ml penicillin. MCF-7, MCF-7/GFP-Mut and K562 cells were grown in RPMI-1640 medium supplemented with 10% FBS, 2 mM l-glutamine, 100 μg/ml streptomycin, and 100 U/ml penicillin. All cells were grown in 5% CO_2_–95% air humidified atmosphere at 37 °C. All media and supplements were obtained from Gibco ThermoFisher Scientific (Waltham, MA, USA).

To evaluate the ligase IV inhibitor and ssODN effect, MCF-7, MCF-7/GFP-Mut, or HCT-116 cells were pretreated with ligase IV inhibitor, SCR7, for 4 h before transfection, and then rinsed with 1 × PBS. For transfection, cells were nucleofected with Nucleofector Solution V (Lonza, Basel, Switzerland) on a Nucleofector (Lonza) using the following programs: P-020 for MCF-7 and MCF-7/GFP-Mut cells, D-032 for HCT-116 cells, and T-016 for K-562 cells. For each nucleofection, 2 × 10^6^ K562 cells, or 1 × 10^6^ HCT-116 or MCF-7 cells in 100 μl of Nucleofector solution were used. The ssODN was dissolved in 10 mM Tris buffer (pH 7.6) to a final concentration of 100 μM, and a 3 μl of this stock solution was mixed with 4 μg of pCS2-Cas9-IRES-GFP-polyA-U6-sgRNA for MCF-7, HCT-116, and K562 cells, or 4 μg of pCS2-Cas9-polyA-U6-sgRNA for MCF-7/GFP-Mut cells before nucleofection. Treatment with ligase inhibitor was continued for another 48 h following nucleofection. At the end of experiment, cells were harvested and rinsed with 1 × PBS. GFP-positive cells were analyzed and sorted by fluorescence activating cell sorter (FACS, BD FACSAria, BD Biosciences, USA).

### Cell cloning

To screen the clones in which ΔTCT mutation of β-catenin Ser45 was corrected, GFP-positive cells were sorted by FACS following HCT-116 cell transfection and 48-h SCR7 treatment. For single-cell cloning, cells were sorted by flow cytometry, and plated to 96-well plates. Notable cell clones were formed after 2-week incubation, and selected clones were expanded for further experiments.

### Genomic DNA isolation, PCR amplification and mutation detection

Genomic DNA was extracted from harvested cells using a DNA isolation kit (Tiangen, Beijing, China). Total 100 ng genomic DNA of each sample was amplified by PCR. To detect whether the *Eco*RI site was inserted into CRISPR–Cas9 targeting site of AAVS1 through HDR, we designed a pair of primers AAVS1-P2 and AAVS1-P4 (Additional file 1: Table S1) for PCR analysis. AAVS1-P2 is an insertion-specific forward primer with GAATTC (*Eco*RI) in the last 6 bases of the 3′ end. GAPDH gene was used as a loading control. PCR was carried out using premix LA Taq with the following cycling condition: for AAVS1-P2P4 amplification: a cycle of 95 °C for 5 min, 35 cycles of 94 °C for 30 s, 62 °C for 20 s and 72 °C for 40 s, and a final cycle of 72 °C for 5 min; for GAPDH amplification: a cycle of 95 °C for 5 min, 27 cycles of 94 °C for 30 s, 62 °C for 20 s and 72 °C for 40 s, and a final cycle of 72 °C for 5 min. Another pair primer, AAVS1-F1 and AAVS1-R1, was used to amplify the CRISPR/Cas9 targeting site of AAVS1, and the PCR products were subjected to TA cloning and DNA sequencing using the following amplification condition: a cycle of 95 °C for 5 min, 32 cycles of 94 °C for 30 s, 60 °C for 20 s and 72 °C for 50 s, and a final cycle of 72 °C for 5 min.

For detection of mutation correction of β-catenin Ser45, we designed two pairs of primers which flank the β-catenin Ser45 site (Additional file 1: Table S1). PCR was carried out using Q5TM Hot Start High-Fidelity 2 × Master Mix (M0494L, New England BioLabs, Ipswich, MA, USA) with the following cycling condition: 98 °C for 1 min, then 36 cycles of 98 °C for 10 s, 59 °C for 20 s and 72 °C for 30 s, and a final cycle of 72 °C for 3 min. The mutation and mutation correction of β-catenin Ser45 was confirmed by DNA sequencing.

### TA cloning and T7 endonuclease I (T7E1) assay

TA cloning was performed by employing pMD18-T vector kit (H101A, Takara, Japan). Targeted gene sequences were amplified using premix LA Taq (Takara). The amplicons were separated using agarose gel electrophoresis and the target bands were extracted and purified using the GEL/PCR Purification Kit (Tiangen, Beijing, China). The DNA concentration was measured with spectrophotometer. For TA cloning ligation, 0.1–0.3 pmol of DNA fragments was mixed with pMD18-T vector and incubated at 16 °C for 1 h or at 4 °C for overnight. The ligation product was transformed into competent *E. Coli* DH5α. Colonies were picked up at next day and plasmid DNA was extracted. The plasmid identify was confirmed by restrict enzyme digestion and DNA sequencing.

For T7E1 reaction, PCR product was denatured, reannealed, and digested with T7 endonuclease I (New England BioLabs Inc, Ipswich, MA, USA), which cleaves mis-matched heteroduplex DNA. After digestion, the PCR product was analyzed by agarose gel electrophoresis [16, 38].

### Restriction fragment length polymorphism assay (RFLP)

Genomic DNA was extracted from transfected cells with the DNA isolation kit (Tiangen, Beijing, China) 3 days after nucleofection. Genomic DNA was then PCR amplified with a pair of primers AAVS1-F3 and AAVS1-R3 flanking the Cas9-AAVS1 target region, which generates a 469/475-bp fragment in 10% acrylamide gel following *Eco*RI digestion. The PCR amplification was carried out with premix LA Taq (Takara, Japan) using the following cycling condition: 95 °C for 5 min for initial denaturation; 32 cycles of 94 °C for 30 s, 55 °C for 30 s and 72 °C for 30 s; and a final extension at 72 °C for 5 min. The cleaved fragment signals were quantified by densitometry using Image J software. The HDR efficiency was calculated as: % = 100 × (1 − (1 − fraction cleaved)^1/2^) [39].

### Clonogenic assay

The clonogenic assay was carried out as previously described [40]. Briefly, HCT-116 parental cells and β-Catenin gene mutation-corrected cell clones were seeded in triplicate in 35-mm plates (500 cells per plate). After 14 days of culturing, cells were stained with Giemsa, and clones containing more than 50 cells were counted. The experiment was repeated four times. The percentage of colonial numbers was calculated by comparison to the control group.

### Western blotting

Cell pellets were lysed in RIPA buffer containing 50 mM Tris (pH 8.0), 150 mM NaCl, 0.1% SDS, 0.5% deoxycholate, 1% NP-40, 1 mM DTT, 1 mM NaF, 1 mM sodium vanadate, and protease inhibitors cocktail (Sigma, St. Louis, MO). Protein concentrations were determined by the Bradford method. Total 40 μg proteins was loaded on 10% SDS-PAGE gels, electrophoresed, and transferred to a nitrocellulose membrane (Millipore, Bedford, MA) [41]. The membrane was blocked by 5% non-fat milk in 1 × TBST (mixture of Tris-buffered saline and containing 0.05% Tween 20) buffer for 1 h at room temperature, and incubated with the primary antibody at 4 °C for overnight. Following washing with 1 × TBST buffer for 10 min for three times, the membrane was incubated with the anti-rabbit or anti-mouse HRP-conjugated secondary antibody (Sigma) for 1 h at room temperature, and the signal was detected using a chemiluminescent western detection kit from Cell Signaling Technology (Beverly, MA, USA). In our experiment, the membrane was first incubated with anti-β-catenin Ser45 phosphorylation antibody (#9564, Cell Signaling Technology). After signal detection, the membrane was stripped off and cut into two pieces at the 70-kd protein marker position. The upper of membrane was then incubated with the anti-β-catenin antibody (#9562, Cell Signaling Technology) for detection the total β-catenin expression, and the lower piece was incubated with the anti-β-actin (Beyotime Biotechnology, China) or anti-GAPDH antibody (G9295, Sigma) as loading controls.

### Statistics

All experiments were repeated at least three times and the data are presented as the mean ± SD. For parametric data, Student’s *t* test was used to determine the statistical significance between two groups, and one-way ANOVA following post hoc Student–Newman–Keuls test was used to compare the difference among multiple groups using the SPSS software. One-side Chi square test was used to analyze the data of HDR efficiency in the presence or absence of ssODN and SCR7 in HCT-116 cells. A p value < 0.05 was considered as statistically significant.

## Results

### The use of Cas9/sgRNA and eGFP co-expression vector together with GFP-positive cells sorting efficiently improved the targeted disruption rate compared to unsorted cells

**Fig. 1:**
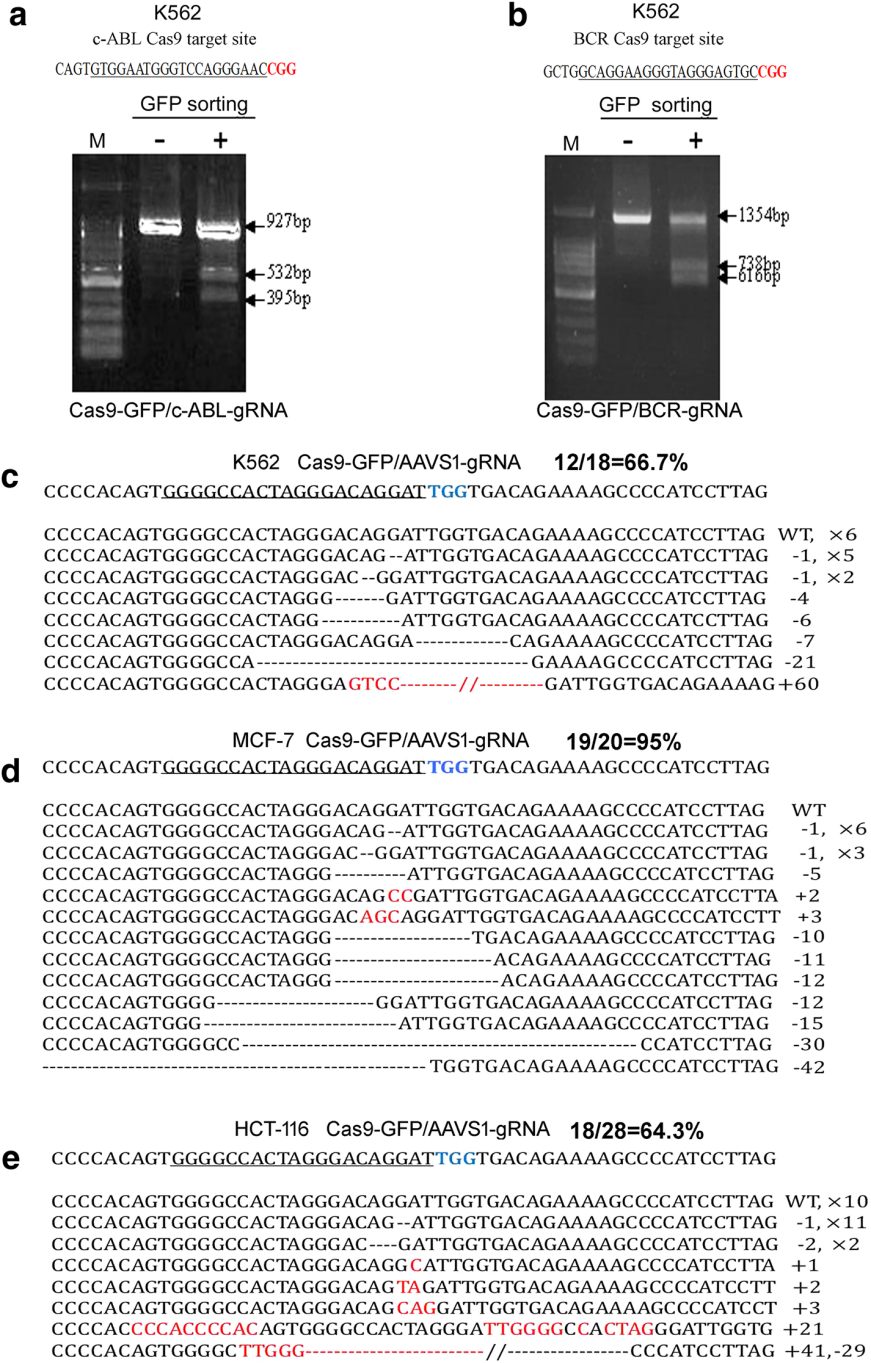
The disruption rates induced by transfecting Cas9, eGFP and sgRNA co-expression vector for three genomic loci in different cancer cell lines. **a** and **b** show the disruption efficiency induced by transfecting Cas9, eGFP and sgRNA co-expression vector in c-ABL and BCR gene loci in K562 cells as determined by T7E1 assay. “−” represents cells without GFP sorting; “+” represents cells with GFP sorting; and “M” represents DNA size marker. **c**–**e** show the disruption rate induced by transfecting Cas9, eGFP and AAVS1-sgRNA co-expression vector in K562, MCF-7 and HCT-116 cells using TA cloning and sequencing analysis. Approximately 20–30 TA clones were randomly picked up for DNA sequencing, and the disruption rate (%) was calculated based on DNA sequencing. All cells were transfected with 4 μg of pCS2-Cas9-IRES-GFP-polyA-gRNA (AAVS1) co-expression vector. Seventy-two hours after transfection, GFP-positive cells were enriched through FACS sorting and genomic DNA was extracted from the sorted cells. The PCR amplification, T7E1 assay and TA cloning into PMD18-T vector were performed as described in “Methods”

To improve transfection efficiency, we assembled Cas9, gRNA and eGFP into one expression vector pCS2-Cas9-IRES-GFP-polyA-sgRNA (Additional file 1: Figure S1). Using this co-expression vector, we are able to trace the transfected cells and enrich the GFP cells by cell sorting. We tested if the co-expression vector together with cell sorting could improve gene targeted disruption efficiency in different cell lines. Firstly, two Cas9 target sites at both BCR and c-ABL genes in K562 cells were used for the detection of the disruption efficiency by T7E1 method. Seventy-two hours following transfection of the co-expression vector, GFP-positive cells were collected through FACS sorting and the genomic DNA was extracted from these GFP-positive cells. The PCR product was denatured, reannealed and digested with T7 endonuclease I. Compared to cells without sorting, the disruption rate in the sorted cells was increased due to enriched transfected cells using GFP-positive sorting as shown in Fig. 1a, b.

To further confirm the efficiency of using co-expression vector together with GFP-positive cell sorting, another method to assay targeted disruption rate after GFP-sorting by TA cloning and sequencing was used at AAVS1 gene loci in three cancer cell lines. The specific fragments were amplified by PCR from genomic DNA of GFP-positive cells and cloned into PMD18-T vector for TA cloning. The disruption rate was defined by TA cloning and DNA sequencing through randomly selecting 20 or 30 clones [16]. Similar to a previous report [7], the disruption rate was approximately 26% when K562 leukemic cells were co-transfected with two separate expression vectors, Cas9 and AAVS1-sgRNA, without cell sorting. However, when K562 cells were transfected with the pCS2-Cas9-IRES-GFP-polyA-gRNA(AAVS1) co-expression vector and the GFP-positive cells were sorted, the disruption rate reached 66.7% as shown in Fig. 1c. A high disruption rate was also obtained in MCF-7 (95.0%) and HCT-116 cells (64.3%) using the same strategy (Fig. 1d, e). These results suggest that the disruption rate could be efficiently improved through enriching targeted cells using the modified co-expression vector together with GFP-positive cells sorting, which will be useful for gene editing of hard-transfecting cell lines.

### SCR7 promoted insertion repair efficiency at AAVS1 locus using ssODN as repair templates in MCF-7 and HCT-116 cells

**Fig. 2:**
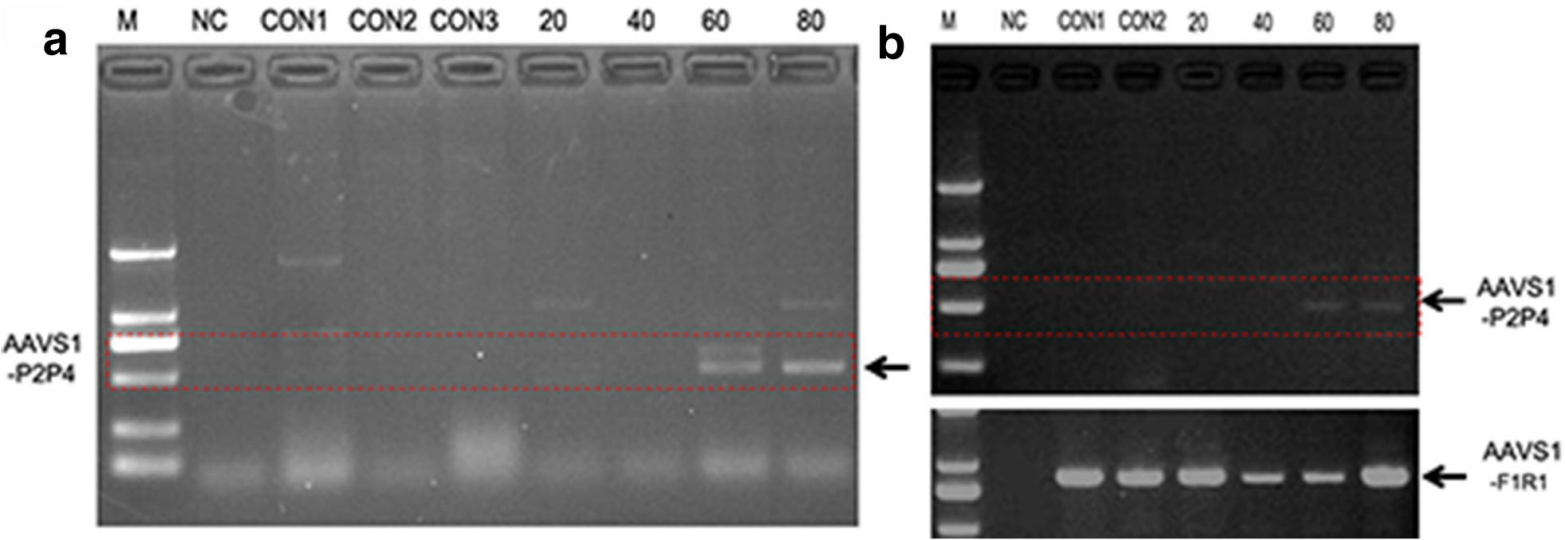
Effect of ssODN homology length on insertion efficiency at the AAVS1 locus. K562 cells were nucleofected with 4 μg of pCS2-Cas9-IRES-GFP-polyA-gRNA (AAVS1) co-expression vector and 0.3 nmol of ssODN donors with different lengths. Cells were harvested 2-day post nucleofection, and the GFP-positive cells were sorted. Genomic DNA was isolated and 100 ng DNA was used for PCR amplification with a P2P4 primer pair (**a**), or P2P4 and F1R1 primer pairs (**b**). The numbers on the top of the gel images represent the homology length in nucleotides of a ssODN donor. A 20-mer donor has two 10-base homology arms. Each ssODN contains an *Eco*RI site between the homology arms. The DNA sequence of each ssODN is shown in Supplementary Table 1. M: DNA Marker. NC: PCR control; CON1: normal cells; CON2: cells transfected with pCS2-Cas9-IRES-GFP-polyA-gRNA (AAVS1) co-expression vector only; CON3: cells transfected with ssODN donor (80 nucleotides) only; 20–80: cells transfected with pCS2-Cas9-IRES-GFP-polyA-gRNA (AAVS1) co-expression vector plus various lengths of ssODNA donors. Arrows indicate the predicted amplified fragments

**Fig. 3:**
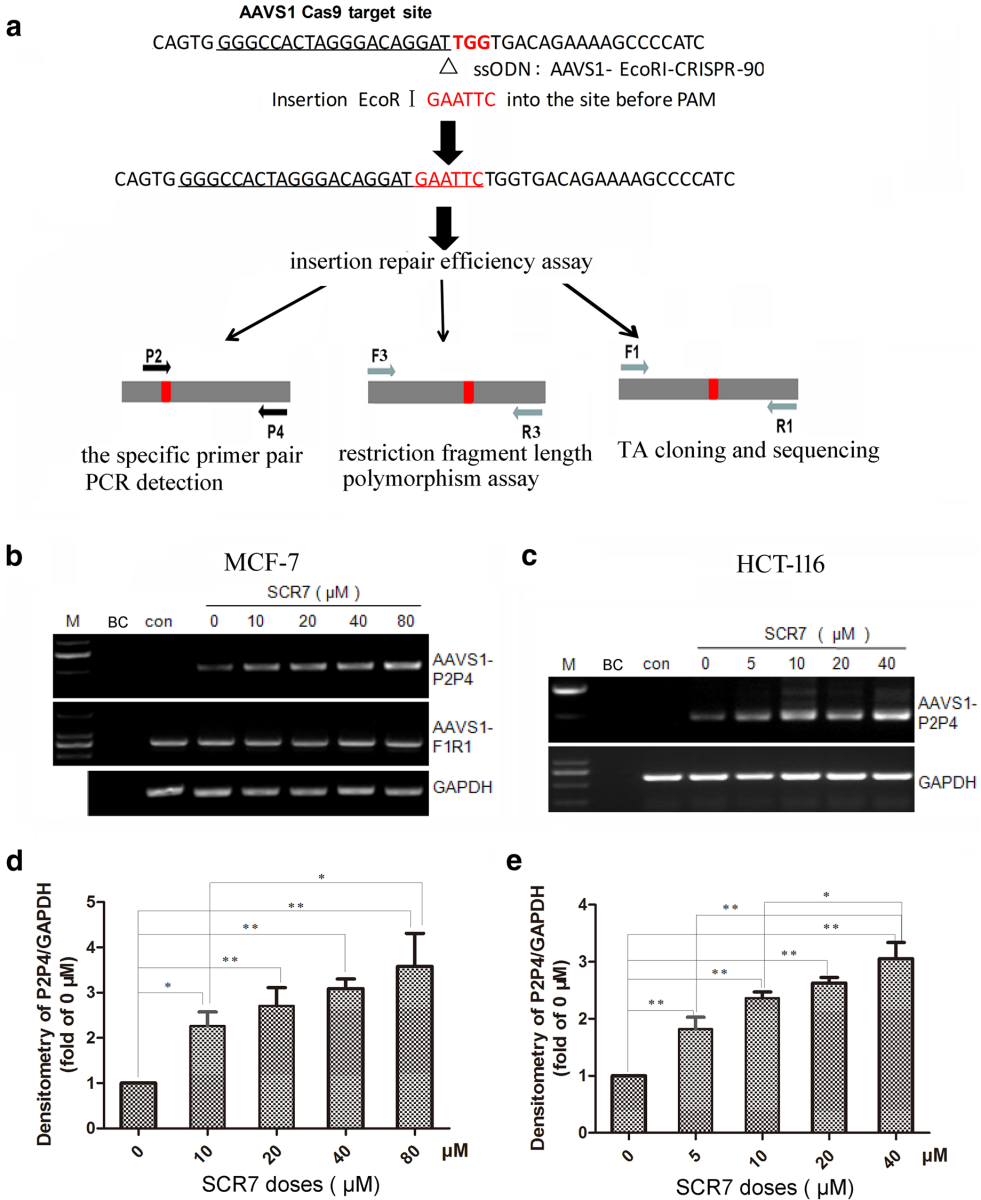
SCR7 promoted insertion repair efficiency at AAVS1 locus. **a** schematically illustrates the insertion repair mediated by ssODN and CRISPR/Cas9 for AAVS1, and the three methods used for the detection of insertion repair efficiency. A pair of primer P2 and P4 were used to examine insertion repair occurred in the Cas9-targeted locus by semi-quantitative PCR-gel electrophoresis. The two pairs of primers F3 and R3, F1 and R1 were used to amplify the sequences involved in the targeted site. The PCR-amplified products were analyzed by RFLP assay following *Eco*RI digestion and by TA cloning and DNA sequencing. **b** and **c** show representative images of PCR amplification with P2 and P4 primer and gel electrophoresis from at least three independent experiments. The data shows the enhancement of insertion repair by ssODN and SCR7 treatment in MCF-7 and HCT-116 cells in a SCR7 dose-dependent manner. “M”—DNA markers, “BC”—blank control without cells, “Con”—control cells without transfection of ssODN and CRISPR/Cas9 vector. **d** and **e** show the quantitative data of PCR-gel electrophoresis analysis using Image J software. GAPDH was used as an internal control. The data is the mean ± SD of three independent experiments. *p < 0.05, **p < 0.01 compared to the corresponding vehicle control

Single-stranded oligonucleotides (ssODN) can be used as templates to repair site-specific DSBs in mammalian cells [15, 25–27]. To test if DNA ligase IV inhibitor, SCR7, can promote insertion repair efficiency at AAVS1 locus using ssODN as repair templates in MCF-7 and HCT-116 cells, a 96-nucleotide fragment, AAVS1-*Eco*RI-CRISPR-96, which contains an *Eco*RI restriction site flanked by 45 nucleotides of homology on each side to the PAM, was directly inserted at a CRISPR/Cas9 cleavage site in the AAVS1 locus (Fig. 3a and Additional file 1: Table S1). To determine the minimal length requirement of ssODN homology, we generated various lengths of homologous AAVS1 ssODNs ranging from 20 to 100 nucleotides (Additional file 1: Table S1), and found that if the length of ssODN was less than 60 nucleotides, the efficiency of HDR was greatly reduced (Fig. 2a, b), suggesting a length-dependent efficacy as previously reported [42]. Since it is difficult to synthesize ssODN above 100 nucleotides in length, a ssODN with 96 nucleotides was optimized as the repair template for our current study (Additional file 1: Table S1).

To minimize the toxic effect of SCR7, we analyzed the dose–response effect of SCR7 on viable cell numbers and observed that SCR7 had an IC50 of approximately 50 and 40 μM in MCF-7 and HCT-116 cells, respectively, when cells were treated for 72 h, which is similar to a previous report [37].

**Fig. 4:**
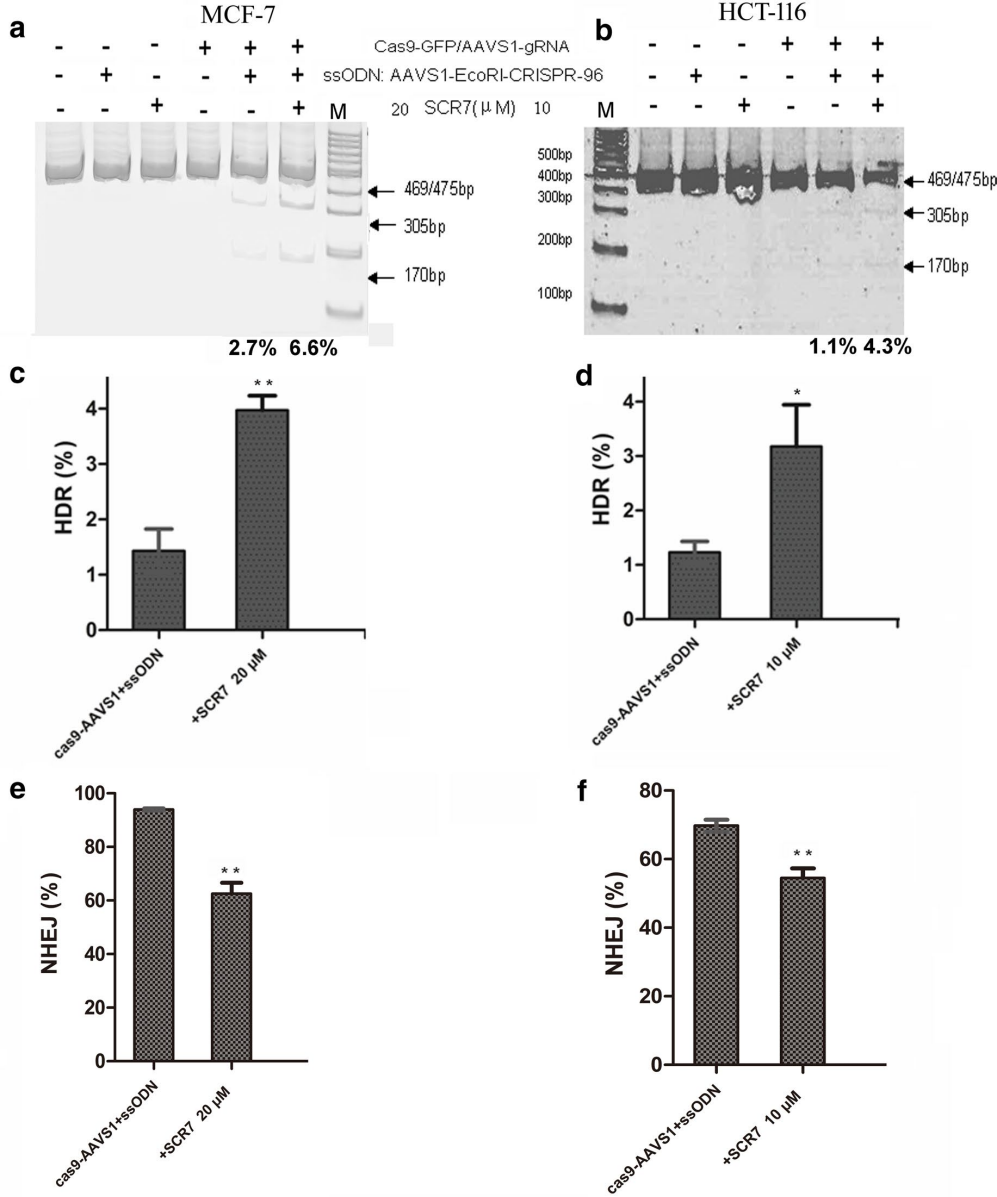
Determination of insertion repair efficiency and NHEJ rate at AAVS1 locus by restriction fragment length polymorphism assay, TA cloning and DNA sequencing. **a** and **b** show the enhancement of insertion repair by ssODN and SCR7 treatment in MCF-7 and HCT-116 cells as determined by restriction fragment length polymorphism assay. Genomic DNA was amplified by PCR, digested with *Eco*RI, and resolved in a 10% acrylamide gel. The original and cleaved DNA fragments are marked by arrows; the signals were quantified by densitometry; and the percentages of the cleaved fragments were calculated as described in “Methods”. **c** and **d** show the quantitation of insertion repair efficiency (%HDR), and Panels **e** and **f** show the percentages of NHEJ at AAVS1 locus by DNA sequencing of TA clones in MCF-7 and HCT-116 cells, respectively. Approximately 100 TA-clones were randomly picked up for DNA sequencing, and the insertion repair efficiency (%HDR) and NHEJ rate (%) in MCF-7 and HCT-116 cells was calculated based on DNA sequencing as described in the Methods. The data are the mean ± SD of three independent experiments. *p < 0.05, **p < 0.01 compared to the corresponding group without SCR7 (*t* test)

Three methods were employed to assess the HDR rate when ssODNs were used as repair templates and combined with a ligase IV inhibitor, SCR7, treatment after transfection of Cas9/gRNA (AAVS1)-GFP vector into MCF-7 and HCT-116 cells as shown in Fig. 3a. First, a pair of specific primers P2 and P4 (Fig. 3a and Additional file 1: Table S1) were used to detect the HDR efficiency. When cells were treated with SCR7, SCR7 greatly enhanced ssODN-directed insertion of target fragment in both MCF-7 (Fig. 3b, d) and HCT-116 cells (Fig. 3c, e). This SCR7 effect was observed at doses as low as 10 and 5 μM and maintained at doses up to 80 and 40 μM in MCF-7 and HCT-116 cells, respectively. Second, the PCR products amplified by AAVS1-F3 and AAVS1-R3 primers (Fig. 3a and Additional file 1: Table S1) were incubated with endonuclease *Eco*RI to cut the HDR fragments. Compared to those without SCR7 treatment, the HDR fragments were obviously increased when cells were treated with SCR7 in both MCF-7 (6.6% vs 2.7%) as shown in Fig. 4a and HCT-116 cells (4.3% vs 1.1%) in Fig. 4b. Finally, to further confirm the target insertion, DNA extracts from GFP-positive cells were amplified by PCR using a pair of primers, AAVS1-F1 and AAVS1-R1, which flank the CRISPR/Cas9 targeting site (Fig. 3a and Additional file 1: Table S1), and the PCR products were subjected to TA cloning and DNA sequencing. Approximately 100 TA clones were randomly selected for sequencing to confirm the targeted insertion of *Eco*RI site as well as the NHEJ clones (Additional file 1: Figure S2). Cells treated with SCR7 showed an approximate threefold increase in targeted insertion efficiency compared to those without SCR7 treatment in both MCF-7 (Fig. 4c) and HCT-116 cells (Fig. 4d) with a significant decrease in NHEJ rate as shown in Fig. 4e, f, respectively. Meanwhile, the rate of wild-type sequence increased accordingly.

These data collectively indicate that SCR7 treatment could increase targeted insertion efficiency of *Eco*RI site in MCF-7 and HCT-116 cells when co-transfected with ssODN and the Cas9/AAVS1-sgRNA expression vector.

### SCR7 enhanced mutation correction efficiency in MCF-7 and HCT-116 cells

**Fig. 5:**
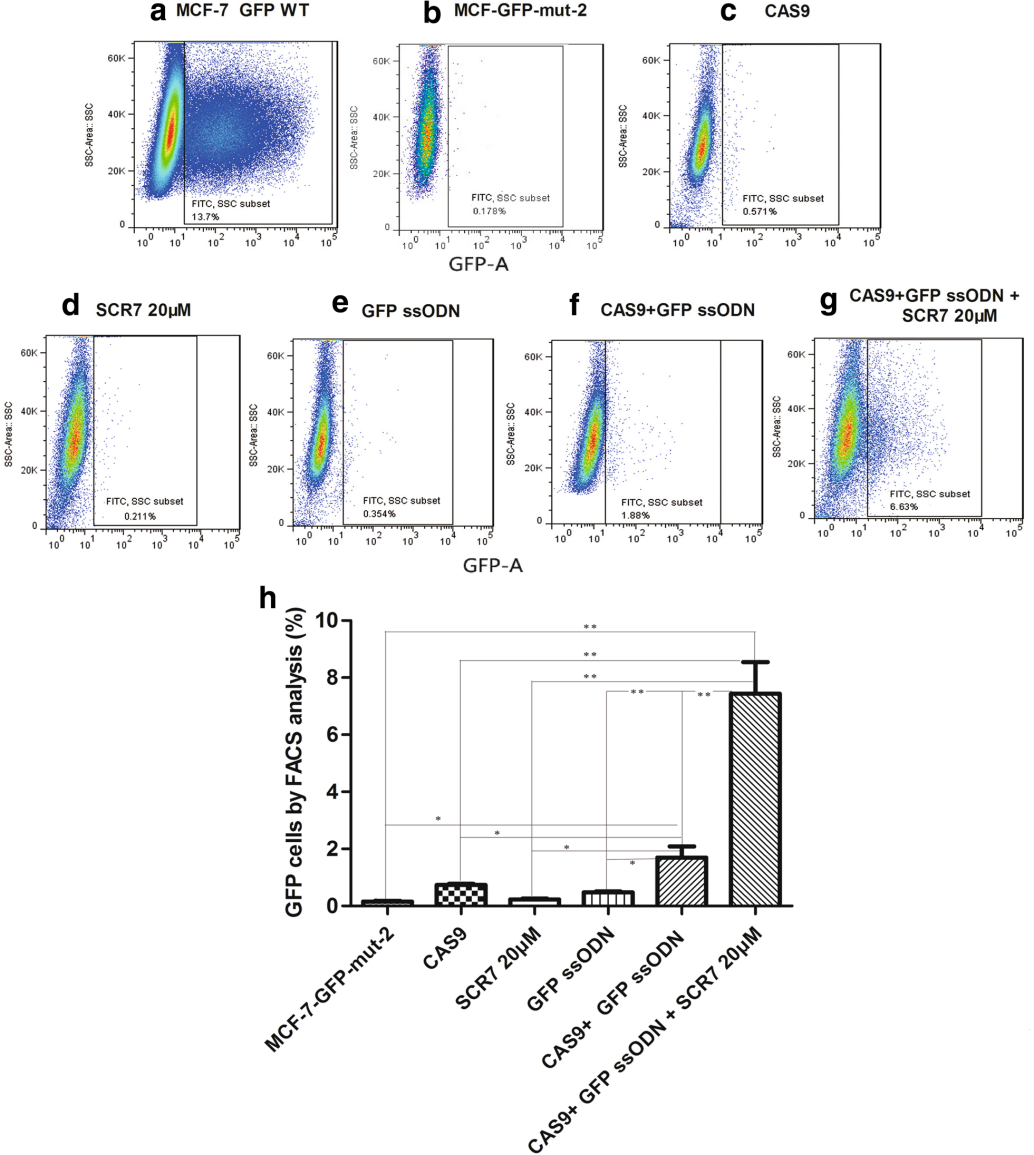
The enhancement of mutation-correction efficiency of a GFP-silent mutation by ssODN and SCR7 treatment in MCF-7/GFP-Mut cells. MCF-7/GFP-Mut cells were co-transfected with the pCS2-Cas9-U6-sgRNA (GFP-Mut) vector or/and GFP ssODN. After transfection, cells were treated with vehicle control or SCR7 (20 μM) for 48 h. At the end of experiment, the GFP-positive cells were quantified by FACS. MCF-7 cells transfected with the wild-type GFP vector (pSIN-EF1-GFP-puromycin) were used as GFP-positive control cells. **a**–**g** show the representative flow cytometric analyses of MCF-7 cells transfected with the wild-type GFP vector (**a**), MCF-7/GFP-Mut cells without transfection (**b**), MCF-7/GFP-Mut cells transfected with pCS2-Cas9-U6-sgRNA (**c**), or GFP ssODN alone (**e**), MCF-7/GFP-Mut cells treated with 20 μM SCR7 alone (**d**), and MCF-7/GFP-Mut cells transfected with both pCS2-Cas9-U6-sgRNA and GFP ssODN without (**f**), or with 20 μM SCR7 treatment (**g**). **h** shows a quantitation of GFP-positive cells in percentage (mean ± SD) from 3 independent experiments. SSC—side scatter. *p < 0.05, **p < 0.01

To determine if SCR7 can improve the efficiency of mutation correction mediated by CRISPR/Cas9 and ssODN, we first constructed a silent mutation in GFP-ORF (GFP-Mut) and established a MCF-7/GFP-Mut-2 cell line through lentivirus-mediated infection (Additional file 1: Figure S3). This MCF-7/GFP-Mut-2 cell line had a very low background of GFP signal due to the GFP-silent mutation (Fig. 5b and Additional file 1: Figure S3B). When these MCF-7/GFP-Mut-2 cells were transfected with the GFP ssODN and a pCS2-Cas9-U6-sgRNA vector that specifically corrects the GFP-silent mutation, the number of GFP-positive cells detected by cell sorting was greatly increased as shown in Fig. 5f, h, indicating a restoration of wild-type GFP signaling. Most importantly, SCR7 treatment resulted in a further improvement of GFP-positive cell expression from 1.9 to 6.6% (Figs. 5f–h), suggesting that SCR7 can improve the CRISPR–Cas9 directed mutation correction.

**Fig. 6:**
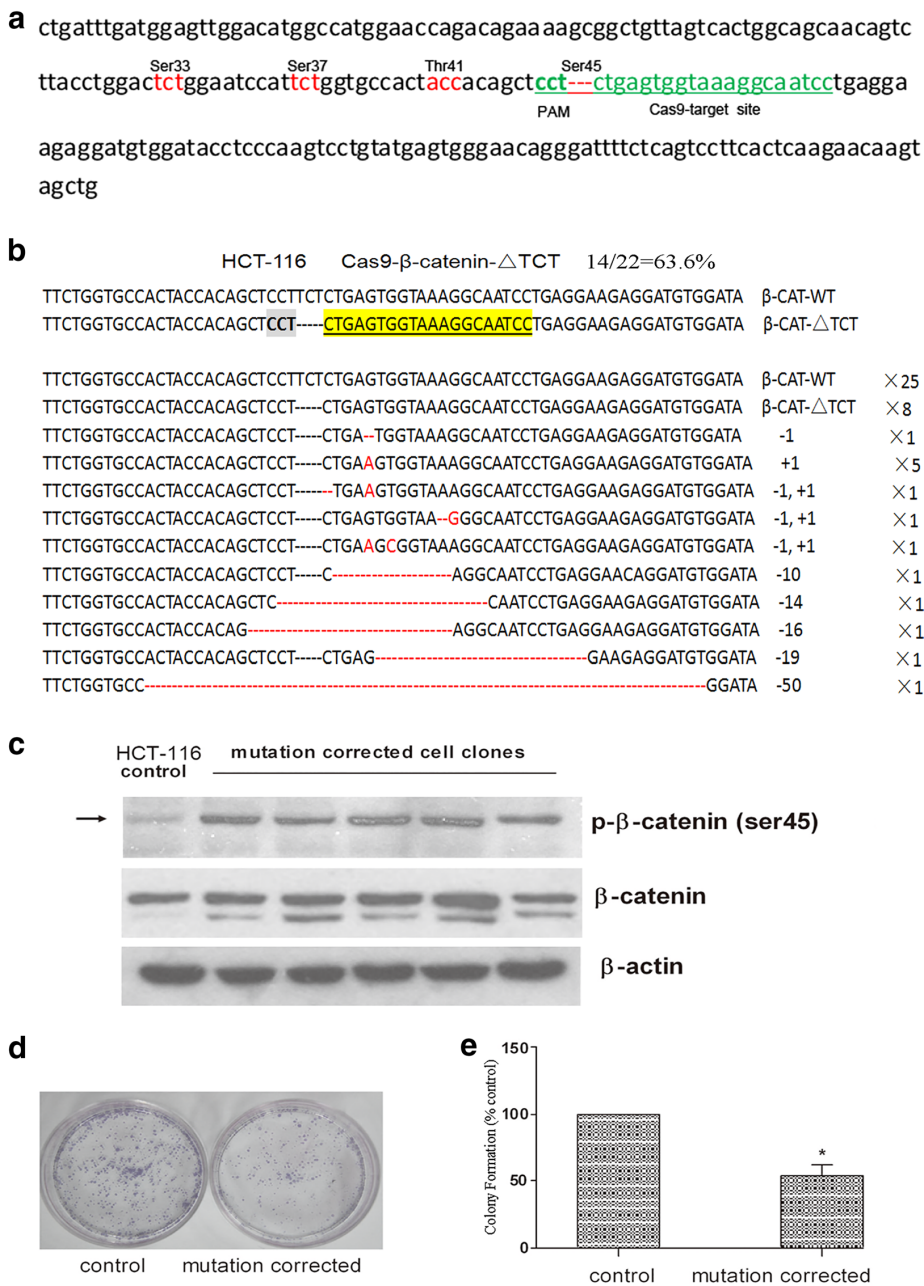
Functional analysis of CRISPR/Cas9-directed mutation correction of a β-catenin Ser45 mutation in HCT-116 cells. **a** depicts the CRISPR/Cas9 targeted mutation site (ΔTCT Ser45) in green letters and surrounding sequences in exon 3 of β-catenin gene. Multiple serine and threonine genetic codes around the target site are marked in red-color letters. **b** illustrates the disruption rate induced by transfecting the Cas9-eGFP-β-catenin-gRNA (ΔTCT Ser45) co-expression vector in HCT-116 cells. **c** shows a representative Western blot analysis of β-catenin Ser45 phosphorylation [p-β-catenin (ser45)] and total β-catenin expression in the parental and multiple mutation-corrected HCT-116 cell clones. β-actin was used as an internal control. **d** shows a representative image of colony formation assay for the parental control and a mutation-corrected HCT-116 cell clone. **e** is a quantification of colony formation in parental control and mutation-corrected HCT-116 cells. Colony formation is presented as percentage normalized to the parental control. The data is the mean ± SD of 4 independent experiments. *p < 0.05 compared to the parental control

Furthermore, we investigated if SCR7 can also improve the efficiency of CRISPR–Cas9 directed mutation correction in cells with a specific endogenous gene mutation. We selected HCT-116 cells that contain a heterozygous Ser45 deletion (ΔTCT) in β-catenin gene [43]. A specific sequence in the deletion mutation allele (ΔTCT Ser45) of β-catenin gene was selected as a CRISPR/Cas9 target (Fig. 6a), and a Cas9/β-catenin-sgRNA and eGFP co-expression vector was constructed and transfected into HCT-116 cells. The disruption rate for the mutant β-catenin gene was approximately 63.6% (Fig. 6b) in the transfected cells following GFP-positive cell sorting.

Due to a deletion mutation in one allele of the gene, the sequencing map of β-catenin in HCT-116 cells showed overlapped peaks starting from the mutation locus (Additional file 1: Figure S4A). To correct the β-catenin gene mutation (ΔTCT Ser45) in HCT-116 cells, a β-catenin wild-type ssODN, β-catenin-WT-96, was prepared as a repair template (Additional file 1: Table S1). When HCT-116 cells were co-transfected with the Cas9/β-catenin-gRNA-eGFP co-expression vector with or without β-catenin-WT-96 ssODN, three outcomes, mutation corrected, HDR without mutation correction, and no HDR are predicted (Additional file 1: Figures S4B–S4D). The cell clones with HDR were judged by the presence of a TCT sequence in the mutation locus. The sequences of mutation-corrected cell clones showed not only that the gene mutation (ΔTCT Ser45) were corrected, but also that the overlapped peaks starting from the mutation locus was disappeared (Additional file 1: Figure S4B). However, the overlapped peaks in the sequencing map was present after the mutation locus in cell clones with HDR but without mutation correction (Additional file 1: Figure S4C). The sequencing data of cell clones without HDR was shown in Additional file 1: Figure S4D.

As shown in Table 1, the efficiency of HDR and mutation correction in co-transfected cells with vector and ssODN was noticeably increased compared to those transfected with the vector alone (8.4% vs 3.7% for HDR, and 7.1% vs 2.8% for mutation correction). Moreover, the addition of SCR7 (10 μM) further enhanced the rate of HDR and mutation correction from 8.4 and 7.1 to 14.6 and 13.9%, respectively. Interestingly, cells transfected with the vector alone without the wild-type β-catenin-WT-96 ssODN also showed a low level of HDR and mutation correction, which was also slightly enhanced by SCR7 treatment (Table 1).

**Table 1:** Efficiency of HDR and mutation correction in HCT-116 cells transfected with a Cas9 co-expression vector in the presence or absence of ssODN and SCR7

Donor	Groups	Rate of HDR (%)	Correction (%)
No donors	Cas9	4/107 (3.7)	3/107 (2.8)
	Cas9 + SCR7	8/114 (7.0)	7/114 (6.1)
β-cateninWT-96	Cas9 + ssODN	13/155 (8.4)	11/155 (7.1)
	Cas9 + ssODN + SCR7	22/151 (14.6)*	21/151 (13.9)*

HCT-116 cells were transfected with the pCS2-Cas9-IRES-eGFP-β-catenin-gRNA(ΔTCT) co-expression vector with or without the addition of β-cateninWT-96 ssODN and SCR7 (10 μM) treatment. The rates of HDR and mutation-correction were derived from direct DNA sequencing of GFP-positive cells as described in the methods. These statistical assays were performed by One-side Chi square test

* p < 0.05 compared to the corresponding Cas9 + ssODN group

To functionally assess the correction of β-catenin Ser45 deletion mutation in HCT-116 cells, we directly analyzed the β-catenin Ser45 phosphorylation and cell proliferation in parental and mutation-corrected cells. As shown in Fig. 6c, the levels of Ser45 phosphorylated β-catenin were significantly increased in the mutation corrected cells compared to the parental control. Moreover, the mutation-corrected cells showed a much lower levels of colony formation compared to the parental cells (Figs. 6d, e). Taken together, this data suggests that the β-catenin mutation in HCT-116 cells was efficiently corrected using the modified CRISPR/Cas9 and ssODN system.

## Discussion

NHEJ and homologous recombination are two major DSB repair pathways in mammalian cells. However, the efficiency of HDR is very poor compared to that of NHEJ, and it is imperative to be improved since HDR is essential for targeted gene editing. In the present study, we have combined three strategies to improve HDR efficiency in mammalian cells: the incorporation of an eGFP signal in the CRISPR/Cas9 system to enrich the transfected cells to improve disruption rate by cell sorting, the administration of a DNA ligase IV inhibitor to block NHEJ pathway, and the use of ssODN as repair templates.

Although CRISPR/Cas9 is a powerful tool to induce DSB in genome loci, it is often a bottleneck to introduce two components, Cas9 and sgRNA, into stubborn cells simultaneously [7, 44, 45]. Through the construction of a co-expression vector of Cas9, sgRNA, and eGFP, we were able to enrich the transfected cells and improve the disruption rates of three different specific loci in K562, MCF-7, and HCT-116 cells using either T7E1 assay or TA cloning and sequencing analysis following GFP-positive cell sorting compared to those without sorting cells (Fig. 1). Compared to other reported strategies such as resistance selection or lentivirus mediated infection to improve transfection [13, 46, 47], this strategy is not only simple, immediate, and fast, but also effective at preventing the integration of Cas9 and sgRNA into the genome of mammalian cells.

Secondary, since NHEJ is a predominant and competitive form of HDR to repair DSBs in DNA, we have used SCR7, a DNA ligase IV inhibitor by targeting its DNA binding domain, to inhibit the NHEJ pathway. As expected, SCR7 treatment decreased the CRISPR/Cas9-mediated NHEJ rate in AAVS1 loci in both MCF-7 and HCT-116 cells (Fig. 4e, f). Consistent with several previous in vitro and in vivo studies [2, 31, 34–36], the addition of SCR7 has greatly increased the HDR efficiency, not only the insertion repair efficiency following co-transfection of AAVS1-96 ssODN and CRISPR/Cas9-GFP vector (Fig. 3 and 4), but also the HDR and mutation-correction efficiency in both established stable MCF-7/GFP-mut-2 cells and β-catenin mutated HCT-116 cells (Figs. 5 and 6, and Table 1). Although an inhibition of NHEJ can induce cell apoptosis [37], we have observed minimal effects of SCR7 on cell viability at the doses used to enhance targeted gene modification, which is consistent with the model that the SCR7 enhancement of targeted gene modification is unlikely due to a change in cell viability as previous suggested [37]. Our current data provides further support to the concept that the blockade of the NHEJ pathway with various approaches is able to enhance HDR and targeted gene editing both in vitro and in vivo systems.

It is intriguing that there was a 3.7% HDR and 2.8% gene mutation correction rate in HCT-116 cells transfected with only CRISPR/Cas9-GFP vectors without ssODN donor. This is probably due to the use of the wild-type allele in this heterozygous β-catenin mutation as the repair template once CRISPR/Cas9 induced a specific DSB in the cells as previously suggested [15]. However, it should be noted that the addition of ssODN in the transfection greatly enhanced the HDR and mutation correction rates (Table 1), which were further improved by SCR7 administration. Taken together, our current data clearly indicates that the combination of a modified CRISPR/Cas9 system with ssODN and ligase IV inhibitor could significantly enhance the efficiency of HDR and targeted gene editing, which may possess significant implications in basic biological research and clinical disease management.

The application of this combinatorial approach for targeted gene modification is exemplified by the functional analysis of gene mutation correction in both a GFP-silent MCF-7/GFP-Mut-2 cell line and a β-catenin mutated (ΔSer45) colon cancer cell line, HCT-116 (Fig. 5 and 6, and Table 1). GFP is a color reporter gene that has been applied widely for the determination of genome-editing efficiency [7, 25]. In the present study, we have used CRISPR/Cas9 and ssODN to correct a GFP-silent mutation established in MCF-7 cells. GFP-silent mutation-corrected cells were quantified using flow cytometry by detecting the restored GFP fluorescence expression, which provides a simple assessment of gene-editing efficiency [48]. Using this convenient system, we have successfully demonstrated the enhancement of a CRISPR/Cas9-directed gene modification of a GFP-silent mutation by a combination of modified CRISPR/Cas9 co-expression vector, ssODN, and ligase IV inhibitor (Fig. 5). Moreover, we have investigated the functional restoration of serine phosphorylation and cell proliferation impacted by a β-catenin ΔSer45 mutation in HCT-116 cells. β-catenin is a key mediator in the classic Wnt signaling pathway and plays a critical role in tumor development and progression [49]. Mutations in the β-catenin gene are detected in 10–50% cases of colorectal cancer and are frequently observed in the region harboring the Ser33/37/Thr41 and Ser45 phosphorylation sites. These β-catenin mutations cause a reduction of β-catenin Ser/Thr phosphorylation and a stimulation of cell proliferation [49, 50]. In the present study, we have successfully corrected the β-catenin ΔSer45 mutation present in the HCT-116 cells using a modified CRISPR/Cas 9 system; the efficiency of HDR and mutation correction is obviously improved by the addition of ssODN and ligase IV inhibitor, SCR7 (Fig. 6 and Table 1). This significant improvement in targeted gene editing highlights the potential clinical application of this combinatorial approach in the gene therapy of colorectal cancers with this β-catenin mutation as well as in other genetic defect diseases.

## Conclusions

We have demonstrated via multiple experimental approaches that a combination of a modified CRISPR/Cas9-GFP co-expression vector, ssODN and DNA ligase IV inhibitor can greatly enhance the efficiency of CRISPR/Cas9-directed gene editing, which has potential applications in targeted gene modification and in gene therapy of genetic defect diseases.

## Additional file

**Additional file 1: Figure S1.** A schematic illustration for constructing a Cas9, eGFP and gRNA co-expression vector. **Figure S2.** Determination of insertion repair efficiency at AAVS1 locus by DNA sequencing. Panel A shows a representative DNA sequencing of a TA clone without a mutation induced by Cas9 and insertion repair at the AAVS1 locus. The Cas9 targeted site of the AAVS1 locus is underlined. Panel B shows a representative DNA sequencing that confirms the incorporation of an ssODN-harbored EcoRI site at the targeted position of the AAVS1 locus. The EcoRI site is underlined. Panel C shows a representative DNA sequencing of a TA clone with NHEJ, but without insertion repair at the AAVS1 locus. The mutation sequences induced by Cas9 are underlined. **Figure S3.** The generation and validation of a GFP-silent mutation lentivirus vector and MCF-7/GFP-Mut cells. Panel A shows part of the GFP ORF with a premature termination codon, tGA, through a replacement of two nucleotides by GA in the sequence (GFP-Mut). Panel B shows a representative fluorescence image of 293T cells used for the package of lentivirus by co-transfecting the pSIN-EF1-GFP-Mut-Puromycin (left) or GFP-Wild type control (right) lentivirus vector together with auxiliary plasmids pSPAX2 and pMD2.G. Forty-eight hours after transfection, the supernatants were collected and the transfected 293T cells were examined by fluorescence microscope (5×). Fluorescence signal is undetectable in 293T cells transfected with GFP-Mut vector (left). Panel C shows that the replacement of two nucleotides, ac, in the wild-type, by GA leads to a formation of a termination codon tGA and a change in the PAM sequence. Panel D shows a representative gel image of T7E1 cleavage assay of disruption efficiency in MCF-7/GFP-Mut cells transfected by Cas9 and GFP-Mut sgRNA. **Figure S4.** Schematic diagrams for DNA sequencing of single cell-derived clones. Single GFP+ cell-derived clones were used to analyze homology-directed repair (HDR) and to evaluate the mutation-corrected rate. Genomic DNA from cell clones was PCR amplified, and the PCR products were sequenced directly. Representative DNA sequencing of cell clones are: Panel A – control HCT-116 cells; Panel B – gene mutation corrected cells; Panel C – cells with HDR but without mutation correction; and Panel D – cells without HDR. **Table S1.** PCR primers and oligonucleotides used for cloning sgRNA expression vector, HDR-mediated repair and Cas9 target sites.

## Abbreviations

CRISPR: clustered regularly interspaced short palindromic repeats
DSB: double-strand break
eGFP: enhanced green fluorescent protein
FACS: fluorescence activating cell sorter
HDR: homology-directed repair
NHEJ: non-homologous end-joining
T7E1: T7 endonuclease I
ORF: open reading frame
PAM: proto-spacers adjacent motif
ssODN: single-strand oligonucleotide
sgRNA: single-guide RNA
ZFN: zinc-finger nuclease
TALEN: transcription activator-like effector nuclease
TBST: mixture of Tris-buffered saline (TBS) and Tween 20
RFLP: restriction fragment length polymorphism
