# Supplementary material for: Ligase IV inhibitor SCR7 enhances gene editing directed by CRISPR–Cas9 and ssODN in human cancer cells
Source: Cell Biosci. 2018 Feb 19;8:12. doi: 10.1186/s13578-018-0200-z (PMC5819182; doi:10.1186/s13578-018-0200-z)
Supplement: Supplementary file 1 — Additional file 1: Figure S1. A schematic illustration for constructing a Cas9, eGFP and gRNA co-expression vector. Figure S2. Determination of insertion repair efficiency at AAVS1 locus by DNA sequencing. Panel A shows a representative DNA sequencing of a TA clone without a mutation induced by Cas9 and insertion repair at the AAVS1 locus. The Cas9 targeted site of the AAVS1 locus is underlined. Panel B shows a representative DNA sequencing that confirms the incorporation of an ssODN-harbored EcoRI site at the targeted position of the AAVS1 locus. The EcoRI site is underlined. Panel C shows a representative DNA sequencing of a TA clone with NHEJ, but without insertion repair at the AAVS1 locus. The mutation sequences induced by Cas9 are underlined. Figure S3. The generation and validation of a GFP-silent mutation lentivirus vector and MCF-7/GFP-Mut cells. Panel A shows part of the GFP ORF with a premature termination codon, tGA, through a replacement of two nucleotides by GA in the sequence (GFP-Mut). Panel B shows a representative fluorescence image of 293T cells used for the package of lentivirus by co-transfecting the pSIN-EF1-GFP-Mut-Puromycin (left) or GFP-Wild type control (right) lentivirus vector together with auxiliary plasmids pSPAX2 and pMD2.G. Forty-eight hours after transfection, the supernatants were collected and the transfected 293T cells were examined by fluorescence microscope (5×). Fluorescence signal is undetectable in 293T cells transfected with GFP-Mut vector (left). Panel C shows that the replacement of two nucleotides, ac, in the wild-type, by GA leads to a formation of a termination codon tGA and a change in the PAM sequence. Panel D shows a representative gel image of T7E1 cleavage assay of disruption efficiency in MCF-7/GFP-Mut cells transfected by Cas9 and GFP-Mut sgRNA. Figure S4. Schematic diagrams for DNA sequencing of single cell-derived clones. Single GFP+ cell-derived clones were used to analyze homology-directed repair (HDR) and to [file 13578_2018_200_MOESM1_ESM.doc]

**Additional information**

Ligase IV Inhibitor SCR7 Enhances Gene Editing Directed by CRISPR-Cas9 and ssODN in Human Cancer Cells

Zheng Hu^1,2^, Zhaoying Shi^3^, Xiaogang Guo^4^, Baishan Jiang^5^, Guo Wang^1^, Dixian Luo^2^, Yonglong Chen^3*^, Yuan-Shan Zhu^1,6*^

^1^Departments of Clinical Pharmacology, Xiangya Hospital, Central South University, Changsha410078, Hunan, China; hu48005@163.com; wangguo32@126.com

^2^Translational Medicine Institute, National & Local Joint Engineering Laboratory for High-through Molecular Diagnosis Technology, the First People’s Hospital of Chenzhou, Chenzhou 432000, Hunan, China; [luodixian_2@163.com](mailto:luodixian_2@163.com)

^3^Department of Biology, Guangdong Provincial Key Laboratory of Cell Microenvironment and Disease Research, Shenzhen Key Laboratory of Cell Microenvironment, Southern University of Science and Technology, Shenzhen518055, Guangdong, China; [shizy1988@163.com](mailto:shizy1988@163.com)

^4^Key Laboratory of Regenerative Biology, South China Institute for Stem Cell Biology and Regenerative Medicine, Guangzhou Institutes of Biomedicine and Health, Chinese Academy of Sciences, Guangzhou 510530, Guangdong, China; [xiaogang.guo@sund.ku.dk](mailto:xiaogang.guo@sund.ku.dk)

^5^Institute of Chemical Biology, Guangzhou Institutes of Biomedicine and Health, Chinese Academy of Sciences, Guangzhou 510530, Guangdong, China; [jiangbs@yeah.net](mailto:jiangbs@yeah.net)

^6^Departments of Medicine, Weill Cornell Medical College, New York, NY 10065.

*Correspondence: 212133@csu.edu.cn/yuz2002@med.cornell.edu; [chenyl@sustc.edu.cn](mailto:chenyl@sustc.edu.cn).

**Fig. S1**

**
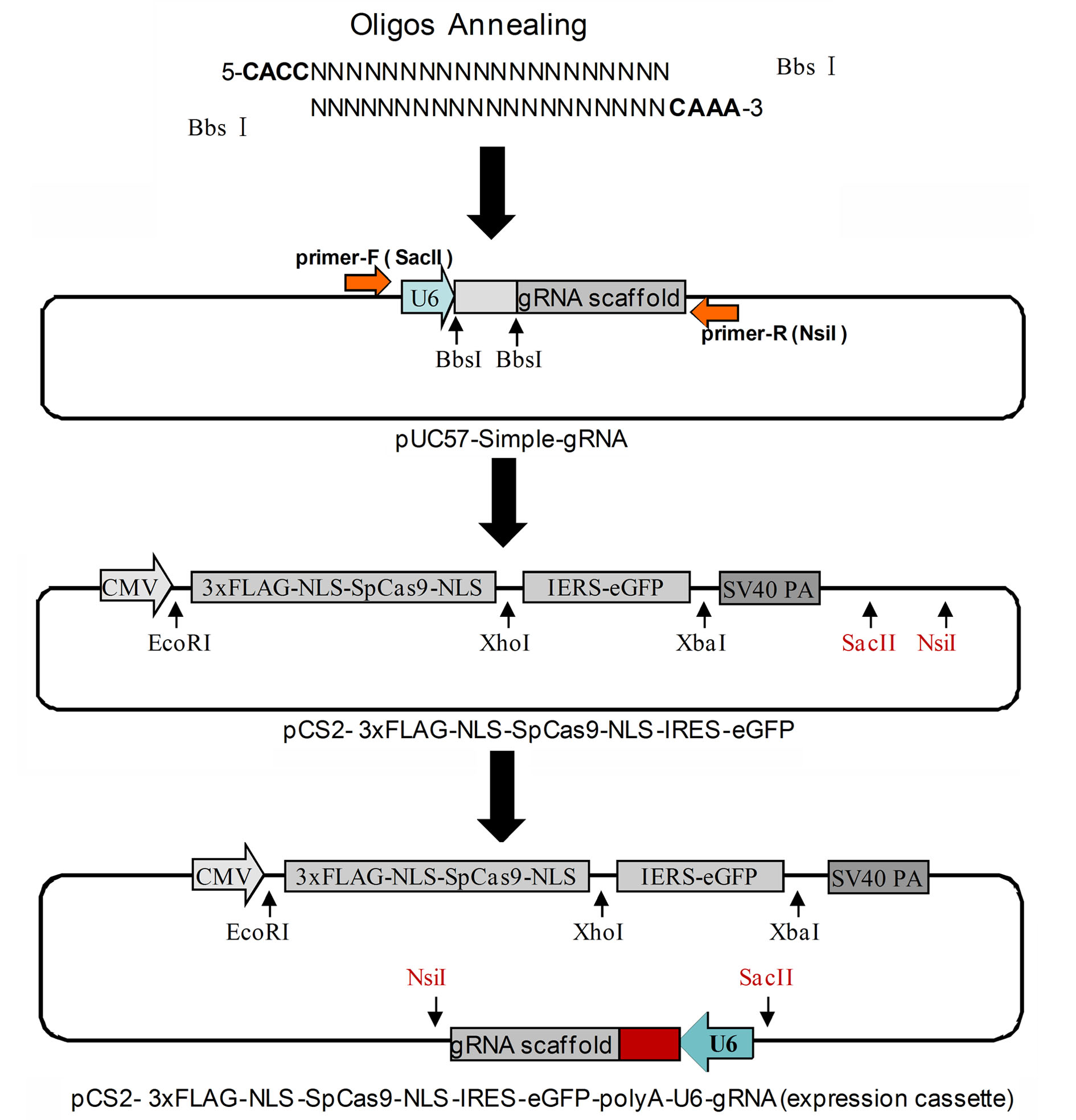
**

**Fig. S1: A schematic illustration for constructing a Cas9, eGFP and gRNA co-expression vector.**

**Fig. S2**

**
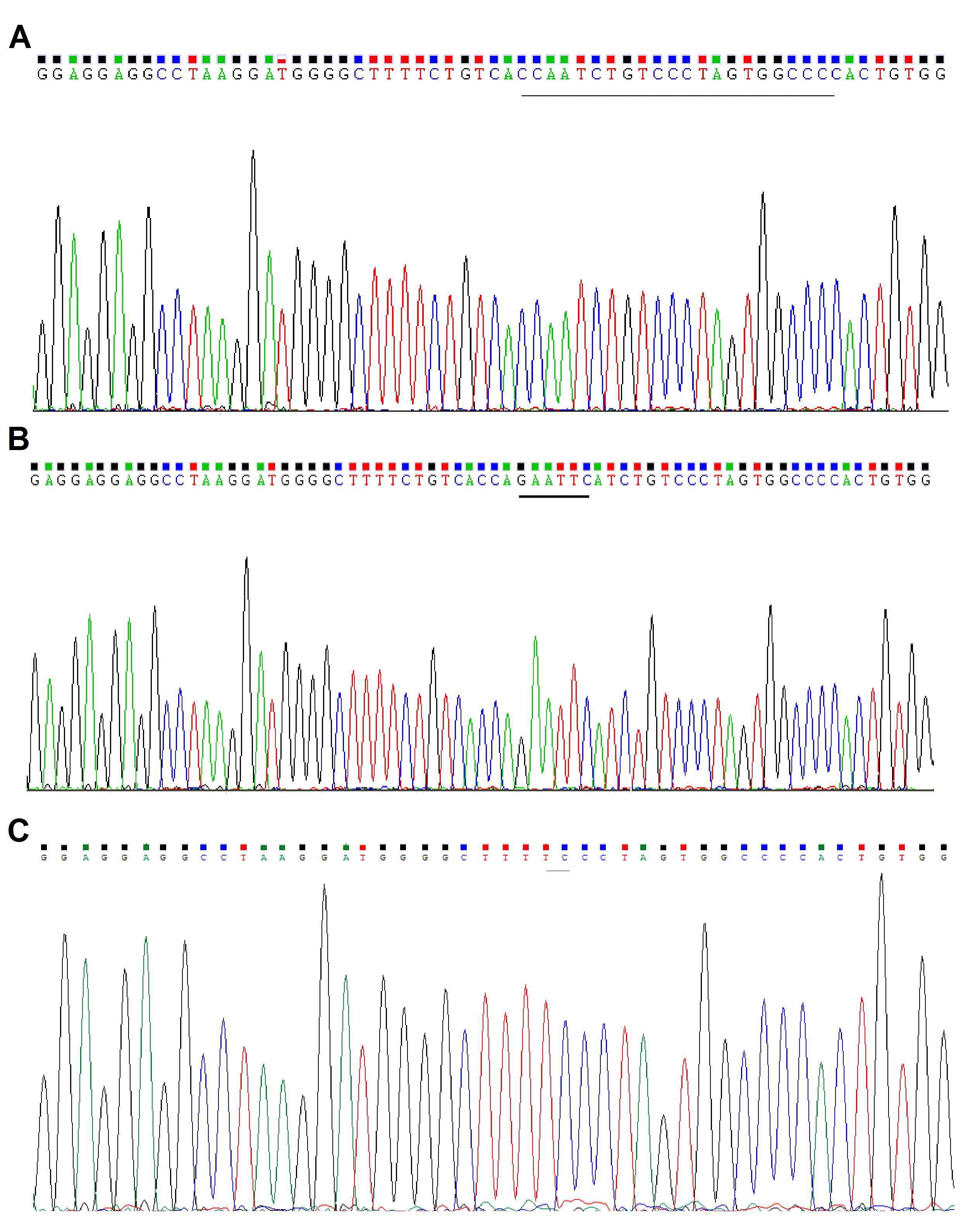
**

**Fig. S2: Determination of insertion repair efficiency at AAVS1 locus by DNA sequencing.** Panel A shows a representative DNA sequencing of a TA clone without a mutation induced by Cas9 and insertion repair at the AAVS1 locus. The Cas9 targeted site of the AAVS1 locus is underlined. Panel B shows a representative DNA sequencing that confirms the incorporation of an ssODN-harbored EcoRI site at the targeted position of the AAVS1 locus. The EcoRI site is underlined. Panel C shows a representative DNA sequencing of a TA clone with NHEJ, but without insertion repair at the AAVS1 locus. The mutation sequences induced by Cas9 are underlined.

**Fig. S3**

**
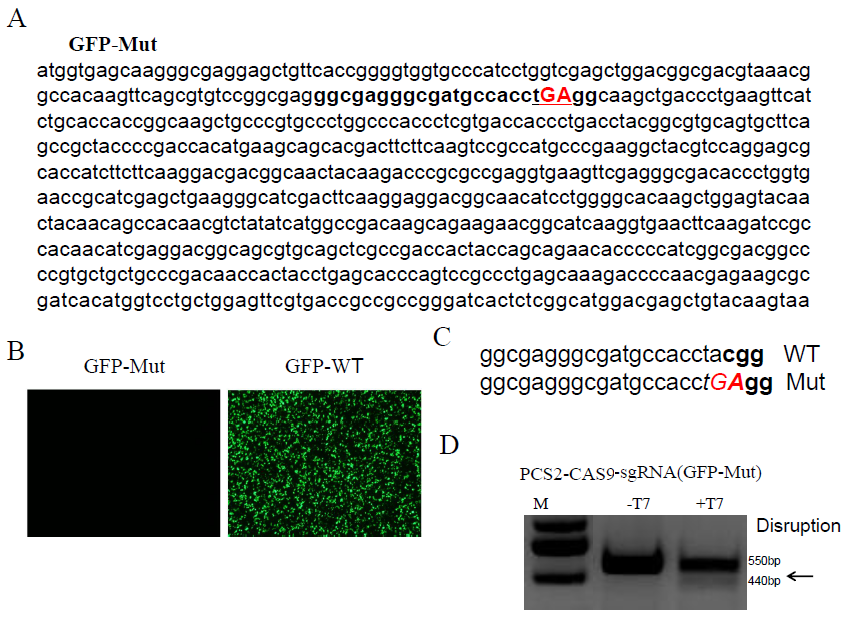
**

**Fig. S3: The generation and validation of a GFP-silent mutation lentivirus vector and MCF-7/GFP-Mut cells.** Panel A shows part of the GFP ORF with a premature termination codon, tGA, through a replacement of two nucleotides by GA in the sequence (GFP-Mut). Panel B shows a representative fluorescence image of 293T cells used for the package of lentivirus by co-transfecting the pSIN-EF1-GFP-Mut-Puromycin (left) or GFP-Wild type control (right) lentivirus vector together with auxiliary plasmids pSPAX2 and pMD2.G. Forty-eight hours after transfection, the supernatants were collected and the transfected 293T cells were examined by fluorescence microscope (5×). Fluorescence signal is undetectable in 293T cells transfected with GFP-Mut vector (left). Panel C shows that the replacement of two nucleotides, ac, in the wild-type, by GA leads to a formation of a termination codon tGA and a change in the PAM sequence. Panel D shows a representative gel image of T7E1 cleavage assay of disruption efficiency in MCF-7/GFP-Mut cells transfected by Cas9 and GFP-Mut sgRNA.

**Fig. S4**

**
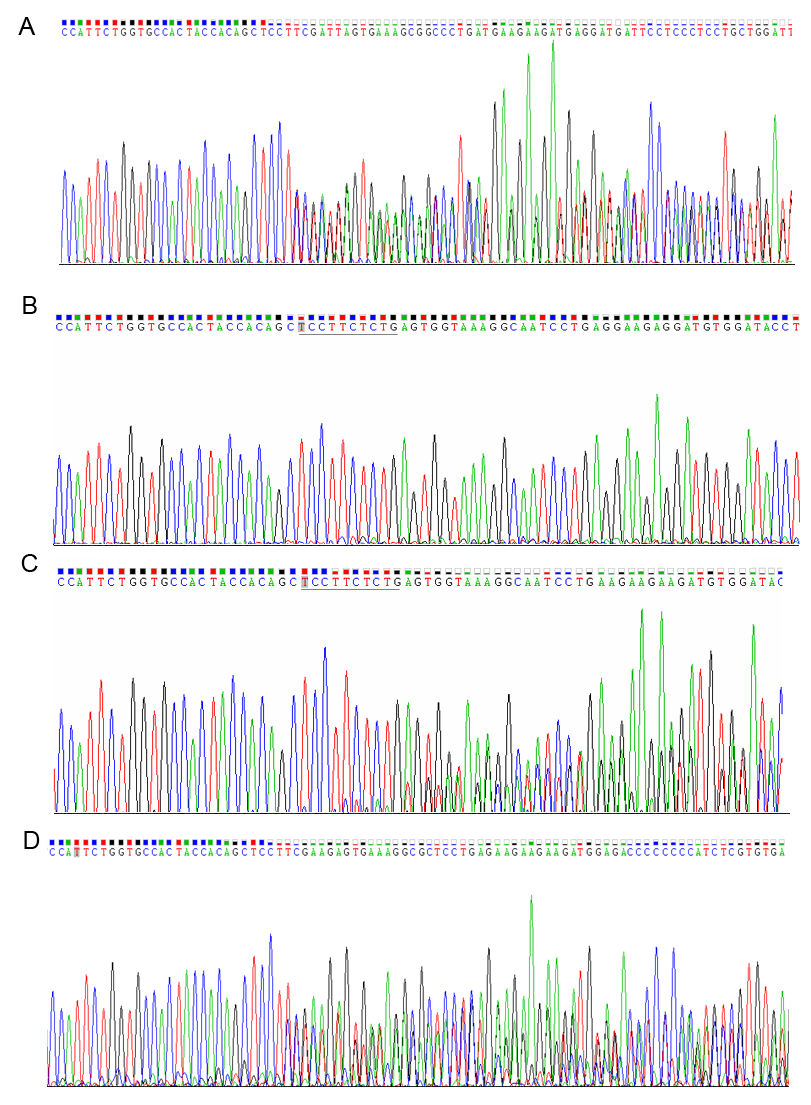
**

**Fig. S4: Schematic diagrams for DNA sequencing of single cell-derived clones.** Single GFP+ cell-derived clones were used to analyze homology-directed repair (HDR) and to evaluate the mutation-corrected rate. Genomic DNA from cell clones was PCR amplified, and the PCR products were sequenced directly. Representative DNA sequencing of cell clones are: Panel A – control HCT-116 cells; Panel B – gene mutation corrected cells; Panel C – cells with HDR but without mutation correction; and Panel D – cells without HDR.

**Table S1: PCR primers and oligonucleotides used for cloning sgRNA expression vector, HDR-mediated repair and Cas9 target sites**

| **Name** | **Sequences (5’ to 3’)** |
| --- | --- |
| BCR-Cas9 target site | GCTGGCAGGAAGGGTAGGGAGTGC**CGG**AAGCGAC |
| c-ABL-Cas9 target site | CAGTGTGGAATGGGTCCAGGGAAC**CGG**CTGAGGA |
| AAVS1-Cas9 target site | CAGTGGGGCCACTAGGGACAGGAT**TGG**TGACAGA |
| GFP Mut-Cas9 target site | CGAGGGCGAGGGCGATGCCACCT**GA**GGCAAGCT |
| β-cateninΔ45S-Cas9 target site | CAGCTCCTCTGAGTGGTAAAGGCAATCCTGAGGA |
| AAVS1-P2 | GGGCCACTAGGGACAGGATGAATTC |
| AAVS1-P4 | AGGTAAAACTGACGCACGGAGGAAC |
| AAVS1-F1 | TCCCTTTTCCTTCTCCTTCT |
| AAVS1-R1 | TTCCAAACTGCTTCTCCTCT |
| AAVS1-F3 | TTCGGGTCACCTCTCACTCC |
| AAVS1-R3 | GGCTCCATCGTAAGCAAACC |
| GAPDH-F | ATGCACTTACCTGTGCTCCC |
| GAPDH-R | GCGCCCAATACGACCAAATC |
| BCR-F | TCCTGGTTGCCTAATGGCAGTG |
| BCR-R | GCACAGTGTGAATGCCCAA |
| c-ABL-F | ACTTTCAAAATACCTGCTGCTTCT |
| c-ABL-R | CGACACTTGAGGAACAAGCC |
| GFP-F | AGCAAGGGCGAGGAGCTGTT |
| GFP-R | TTCTGCTGGTAGTGGTCGGC |
| β-catenin-F1 | CCTGGCTATCATTCTGCTTT |
| β-catenin-R1 | TCAACACTCACTATCCACAG |
| β-catenin-F2 | CTGAGATCCCCCTGCTTTCC |
| β-catenin-R2 | CAGGACTTGGGAGGTATCCAC |
| sgRNA-primer-F(Sac II) | TCCCCGCGGAAGGTCGGGCAGGAAGAGG |
| sgRNA-primer-R(Nsi I) | CCAATGCATAAAAAAAGCACCGACTCGG |
| Xho I-IRES-eGFP-F | CCGCTCGAGGCCCCTCTCCCTCCCCCC |
| Xba I-IRES-eGFP-R | TGCTCTAGATTACTTGTACAGCTCGTCCAT |
| CRISPR-BCR-sense | CACCGCAGGAAGGGTAGGGAGTGC |
| CRISPR-BCR-antisense | AAACGCACTCCCTACCCTTCCTGC |
| CRISPR-c-ABL-sense | CACCGTGGAATGGGTCCAGGGAAC |
| CRISPR-c-ABL-antisense | AAACGTTCCCTGGACCCATTCCAC |
| CRISPR-AAVS1-sense | CACCGGATTGCCTTTACCACTCAG |
| CRISPR-AAVS1-antisense | AAACCTGAGTGGTAAAGGCAATCC |
| CRISPR-GFP-Mut-sense | CACCGGCGAGGGCGATGCCACCTG |
| CRISPR-GFP-Mut-antisense | AAACCAGGTGGCATCGCCCTCGCC |
| CRISPR-β-cateninΔ45S-sense | CACCGGATTGCCTTTACCACTCAG |
| CRISPR-β-cateninΔ45S-antisense | AAACCTGAGTGGTAAAGGCAATCC |
| AAVS1-EcoRI-CRISPR-96 | TTATCTGTCCCCTCCACCCCACAGTGGGGCCACTAGGGACAGGAT**GAATTC**TGGTGACAGAAAAGCCCCATCCTTAGGCCTCCTCCTTCCTAGTCT |
| AAVS1- EcoRI-CRISPR-80 | TGTCCCCTCCACCCCACAGTGGGGCCACTAGGGACAGGAT**GAATTC**TGGTGACAGAAAAGCCCCATCCTTAGGCCTCCTCCTTCCT |
| AAVS1- EcoRI-CRISPR-60 | ACCCCACAGTGGGGCCACTAGGGACAGGAT**GAATTC**TGGTGACAGAAAAGCCCCATCCTTAGGCCT |
| AAVS1- EcoRI-CRISPR-40 | GGGGCCACTAGGGACAGGAT**GAATTC**TGGTGACAGAAAAGCCCCAT |
| AAVS1- EcoRI-CRISPR-20 | GGGACAGGAT**GAATTC**TGGTGACAGA |
| GFP ssODN | GGCCACAAGTTCAGCGTGTCCGGCGAGGGCGAGGGCGATGCCACCTACGGCAAGCTGACCCTGAAGTTCATCTGCACCACCGGCAAGCTG |
| β-cateninWT-96 | CTTACCTGGACTCTGGAATCCATTCTGGTGCCACTACCACAGCTCCTTCTCTGAGTGGTAAAGGCAATCCTGAGGAAGAGGATGTGGATACCTCCC |
